# Displacement of the Coccyx Sideways

**Published:** 1859-09

**Authors:** 


*Displacement of the Coccyx sideways. By Dr. Rœser.* (Prager Vierteljahrschr, Nov. 18th, 1858, and Edinburgh Medical Journal, Nov., 1858.)

*Case.*—"A corpulent woman, aged 36, fell from a table on a chair, so that its back came right between her thighs. She instantly felt severe pain in the coccyx, but continued able to move about till evening, sitting increasing the pain very much. In the evening, the pain was so great, extending up the spine, that she was obliged to go to bed, and soon after could neither turn nor rise up. After a painful night, R—— found this otherwise blooming woman quite immovable, with distorted features; she complained of violent pain in the coccyx, and a painful tensive drawing feeling from below, up to the neck, which also extended down the arm. She could move the forearm a little. The slightest motion of the body or head to one side was impossible, and still more so, sitting up in bed; confused headache, and some mental disturbance, were also present. She made no complaints of her lower extremities, nor of her arms, and urinated without difficulty. After placing her on her right side, a small swelling, the size of a hazel nut, was felt near the notch of the buttocks, next the left ischium, which on closer examination proved to be the coccyx separated from the sacrum, and forced from the mesian line towards the left ascending ramus of the ischium. The obtuse end of the sacrum could easily be felt between the buttocks. By placing one finger in the rectum, the dislocation of the coccyx could be still more easily felt. Forcible pressure downwards and towards the right buttock caused it suddenly to glide into its normal position, whereupon the patient declared herself relieved, herself as if roused from a dream, and all her pains vanished. She could move about freely, but pain in the sacro-coccygeal region prevented her sitting up. Her expression was also completely changed. After a few days, a dull pain in the sacro-coccygeal region, preventing sitting, was all the uneasiness that remained; and in five days, all of this that was left was a slight burning sensation at the injured spot."

"The irritation of the spinal marrow observed in this case, in which only the very lowest filaments could have been disturbed, and which nevertheless sent the stream of disturbance to the brain itself, is a most interesting example of mechanical irritation, as evidenced by its instant disappearance on the reduction of the dislocation."—*Ranking.*

This must be a very rare accident. Many surgeons even deny the possibility of its occurrence, and particularly so after forty years of age. Accidents are by no means unusual in the sacro-coccygeal region, but then, when severe, and the subject past thirty years, it is a fracture, not dislocation, we have to deal with. And as we reflect on the mode of union subsisting between the saocrum and coccyx; on the security provided for, by a strong posterior ligament, by a fibro-cartilage, similar to what exists between the vertebræ, stretching from the base of one to the point of the other, and by a muscular apparatus extending from the coccyx to the ischium, it becomes evident that an injury sufficient to alter the relations of these bones, to such a degree as to cause complete luxation, must be attended not merely with such symptoms as occurred in the case detailed, at the time of, and immediately following the accident, but will most probably give rise to extensive abscess in the loose cellular tissue around the rectum. The ramifications of the middle sacral artery are in almost all such cases ruptured, and the blood effused generally leads to suppuration. Even where there is neither fracture nor dislocation, contusions in this region often induce grave symptoms. Though the contusion leaves no visible traces—no ecchymosis—a pain darting from the point of the sacrum extends to the sacral and femoral regions, attended with great suffering in defecation and urination. About the fifth to the seventh day, a rigor takes place, with a sense of fullness and pulsation in the part. An abscess forms, on opening which we find the rectum denuded, and most probably, also, necrosis of the coccyx.

